# Suicidality Presented to a Child and Adolescent Psychiatry Emergency Service: Increasing Rate and Changing Characteristics

**DOI:** 10.3389/fpsyt.2021.708208

**Published:** 2021-07-15

**Authors:** Stephanie Kandsperger, Irina Jarvers, Daniel Schleicher, Angelika Ecker, Michael Wirth, Romuald Brunner

**Affiliations:** Clinic of Child and Adolescent Psychiatry, Psychosomatics and Psychotherapy, University of Regensburg, Regensburg, Germany

**Keywords:** suicidal thoughts, suicide attempt, emergency, non-suicidal self-injury, adolescents, children, mental health, crisis intervention

## Abstract

**Background:** Children and adolescents who present to child and adolescent psychiatric emergency departments show a variety of reasons for their presentations. Suicidality, in particular suicidal thoughts and suicide attempts, as well as non-suicidal self-injury (NSSI) play a large and important role. In this context, inpatient admissions frequently serve as crisis intervention.

**Methods:** In this study, face-to-face emergency presentations to the emergency department at our Clinic of Child and Adolescent Psychiatry (CAP) were analyzed over the years 2014-2018, the 4th quarter (October-December) of each year. Data from 902 emergency presentations were evaluated, primarily with regard to suicidal thoughts, suicide attempts, and NSSI as reasons for presentation.

**Results:** Data demonstrated that the number of emergency presentations increased in general and especially for suicidal thoughts and NSSI as reasons for presentation. In addition, suicidal thoughts, suicide attempts, and NSSI as reasons for emergency presentation were more likely to result in crisis-related inpatient admissions. Furthermore, reporting suicide attempts at emergency presentation was associated with longer inpatient stays. Finally, cases with multiple diagnoses increased independent of the general increase in emergency presentations.

**Conclusion:** The increase of utilization of clinics with CAP outpatient emergency patients and following admissions to the inpatient units for crisis intervention poses a major challenge for the future. It is important to prepare for the assessment and treatment of suicidality, which is of extraordinary importance in the care of emergency patients.

## Introduction

According to a large epidemiological study 16.9% of children and adolescents living in Germany show mental health problems (1), comparable with rates in other European countries (2). It is of particular relevance that only between 20% (3) and 50% (4) of those affected receive treatment. Many children and adolescents who meet the criteria for mental illnesses have an unmet need for support and especially those affected, even with mild symptoms, should receive prompt treatment (5). It is estimated that about 10% of children and adolescents suffer from their mental disorders to such an extent that they are not only affected on a daily basis, but the disorders can continue into adulthood if they remain untreated (6). Unfortunately, targeted treatment often begins years after the first onset of initial symptomatology (7).

Emergency child and adolescent psychiatric care is often the entry point to mental health care (8, 9). The number of emergency-related child and adolescent psychiatric visits has been growing in recent years (10–12). A study from the largest child and adolescent psychiatric hospital in the Paris area investigated emergency presentations over a long period of time and, as a result, found an annual increase by 3.85 times between 1981 and 2017, with the largest increase in the 1980s and then less pronounced over time (13). Context for emergency presentations varies from minor issues to life-threatening crises (14). Numerous reasons are given for emergency presentations, but most commonly the goal is the assessment of self-injurious thoughts and behaviors (SITB) (15). In addition to non-suicidal self-injury (NSSI), which is carried out without the intention to die, suicidal behavior, in which the person has at least to some degree the intention to die, is defined as SITB (16). Since the intention to die is often times ambivalent in patients, there is an overlap between NSSI and suicidal behavior (17), which is the reason why both aspects should be focused on in an emergency setting. Suicide attempts and NSSI represent distinct behaviors that differ in intent, form, and function, yet the two behaviors frequently occur simultaneously in adolescents as well as adults (18); the same can be concluded for suicides (19).

The prevalence of SITB reported by parents in children younger than 11 years was very low (20). However, in a representative sample of 12,068 adolescents from 11 European countries, an overall lifetime prevalence of direct self-injurious behavior (regardless of suicidal intent) of about 27.6% could be detected (17). The 12-month prevalence of NSSI in a clinical sample of inpatients was even higher with 60% and nearly 50% who engaged in it repeatedly (21). In a representative sample of 44,610 students in the 9th grade of different school types in Germany, the rate of suicide attempts (lifetime prevalence) was 9% and the prevalence of suicidal ideation was 39.4% (5.2% often, 10.4% sometimes, and 23.8% rarely) (22). Suicide is one of the leading causes of death among youth worldwide (23). In this respect, risk assessment but also, in general, clinical assessment of the mental health problems of children and adolescents presenting in an emergency plays a prominent role, including the crucial decision whether to proceed with inpatient admission for crisis intervention (24).

A study in a Clinic of Child and Adolescent Psychiatry (CAP) in Germany showed that utilization through crisis admissions increased by 219% between 2005 and 2015, compared to a simultaneous 23.7% increase in regular admissions (15). However, there are studies in Europe that show a different trend of inpatient admissions after psychiatric emergency consultation in adolescents, for example a study conducted in France (13). Here, admission to an inpatient unit immediately after an emergency consultation accounted for three-quarters of the total annual number of consultations in 1981, but only 15.6% in 2017 (13). In a study from Denmark, the inpatient admission rate decreased from 19.2 to 15.7% between 2003 and 2006 (14). In this context, it would be interesting to examine how the trend in inpatient emergency admissions has developed in recent years.

This present study aimed at investigating the rate and possibly changing demographic and clinical characteristics of the outpatient emergency consultation of our clinic over a time course of 5 years. In particular, we analyzed the reasons given for the presentations with a focus on acute crises in connection with suicidal thoughts, suicide attempts, and NSSI. We expected a significant increase in emergency presentations over time. Furthermore, according to our clinical impression, we anticipated an increase in the presentation of reasons due to suicidal thoughts, suicide attempts, and NSSI over time. We also proposed that these reasons for emergency consultation were more likely to result in crisis-related inpatient admissions. In addition, we generally assumed a higher proportion of cases with multiple diagnoses over time. We also conceived of a higher number of psychiatric diagnoses among emergency presentations that presented for NSSI or suicidality. For example, it has already been shown that substance abuse can have a significant impact on suicidality (25), especially during the adolescent phase (26, 27). Specifically, patients with substance-induced psychosis have been shown to have a higher prevalence of suicidal ideation during the past year (28). In general, but also especially in emergency presentations, possible comorbid diagnoses (e.g., substance disorders) should not be overlooked, as these should be considered in treatment and often manifest in higher symptom severity (29, 30). Analyzing child and adolescent psychiatric emergency presentations is crucial in order to provide sufficient and appropriate resources for adequate emergency care. With additional knowledge of the clientele and the changes in recent years, emergency service in child and adolescent psychiatry can be adapted to future needs.

## Materials and Methods

The Clinic of Child and Adolescent Psychiatry, Psychosomatics and Psychotherapy at the University of Regensburg, Germany, is a typical child and adolescent psychiatric hospital of maximum care. It includes three full inpatient units (40 beds), three day-care units (22 patients) and offers a comprehensive outpatient care unit. The catchment area of our CAP service includes around 178,000 children under the age of 18 years. In this report, face-to-face emergency presentations in the emergency outpatient department 24 h for 7days a week of the years 2014-2018 of the respective fourth quarter are examined, i.e., from October 01 to December 31. The fourth quarter for each year was chosen because clinical experience showed that these are the most demanding months of the year. Thus, a longer time period could be considered and seasonal fluctuations in the frequency of selected mental disorders [e.g., seasonal affective disorders (31)] could be excluded a priori. Patients from rural areas or large and medium-sized cities throughout the Upper Palatinate (Oberpfalz) were treated during regular consultation hours, on weekends and holidays. During regular consultation hours, it serves the area of the city of Regensburg, the districts of Regensburg, Neumarkt and Schwandorf as well as the entire area of the Upper Palatinate after regular consultation hours. The Upper Palatinate is a government district of the Free State of Bavaria and has 1,106,269 inhabitants, with Regensburg being the only major city with 168,876 inhabitants. The sample described here includes patients of the emergency presentations in Regensburg as well as patients of the branch offices for whom an inpatient admission had to be assumed or was already indicated. This results in a sample size of 927 emergency presentations in the observation period, of which 902 were finally included in the data evaluation due to lack of information (for example, no detailed information about the reason for presentation).

The evaluation was based on the charts and electronic documentation records filled in by the physicians on duty and the physicians who continued the treatment. Clinical notes, doctor's letters, outpatient and inpatient treatment documentation stored in the electronic hospital information system were used to evaluate the amount and clinical characteristics of the emergency presentations. The parameters were collected to the best of our ability from the above-mentioned documentation. No specific scales were used, for example, to elicit the presence of suicidal thoughts or suicide attempts, as this was a purely retrospective analysis of existing data. Clinical psychiatric diagnoses were based on chapter V (F) of the International Classification of Diseases (ICD-10). Furthermore, compulsory presentations and the reasons for the presentation (e.g., suicidal thoughts, suicide attempts, non-suicidal self-injury, psychosocial stress factors, and aggression by others) were also obtained in this context.

### Statistical Analyses

To test the relationships between the main variables time (in months), number of emergency presentations (specific to suicidal thoughts, suicide attempts, and NSSI) and number of diagnoses, non-parametric Kendall's τ correlations were calculated for each hypothesis, as the τ coefficient is less susceptible to deviations in distributions and possible outliers. When a Kendall's τ correlation did not show a significant result, an exploratory partial correlation was calculated in reasoned cases in order to control for a possible intervening variable. If a significant correlation with time (in months) was found, it was entered into a simple linear regression as an independent variable in order to compute the trend of cases over time. The relationship between suicidality, NSSI, and inpatient admissions was investigated through Chi-Square tests, particularly whether presenting with suicidality or NSSI as a reason was more likely to result in an inpatient admission than not. Additionally, to investigate whether suicidality and NSSI as reasons for emergency presentations resulted in longer inpatient stays, Mann-Whitney *U*-tests were conducted. Finally, the rate of “cases with multiple diagnoses” over the years was computed, with “cases with multiple diagnoses” defined as cases in which a patient received equal/more than two diagnoses. The rate was used to determine the increase of cases with multiple diagnoses over the years without a bias of a general increase in emergency presentations. In case of a significant correlation, a simple linear regression was computed to determine how much variance in the rate of cases with multiple diagnoses to cases with single diagnose can be explained by time alone. In cases with several tests for a single hypothesis, the false discovery rate (FDR) was used to correct for multiple comparisons (32). Reported *p*-values already correspond to the correction. All major statistical analyses were conducted using SPSS 25 (IBM Corp. Released 2017. IBM SPSS Statistics for Windows, Version 25.0. Armonk, NY: IBM Corp.) and the statistical significance level was set to α = 0.05.

## Results

### Sample

Detailed sociodemographic and clinical characteristics of patients presented to the outpatient emergency service is presented in Table 1. A total of 902 cases of emergency presentations were included in the sample. Children and adolescents up to an age of 18 years, in exceptional cases up to 21 years were included in the study. The average age was about 15 years (*M* = 15.4, *SD* = 2.6). Slightly more than half of the presentations were female (57.8%). Among the reasons for presentation given by the patients, we focused on the reasons suicidal thoughts, suicide attempts, and NSSI. Suicidal thoughts were found to be the most common reason for emergency presentation (*N* = 489, 54.2%). NSSI was given as a reason for presentation by a quarter of emergency presentations (*N* = 231, 25.6%). Suicide attempt was given as reason for presentation in nearly 5% of emergency presentations (*N* = 42, 4.7%). It should be noted here that patients were able to provide multiple reasons for emergency presentations and that some of the patients presented more than once within a quarter. Patients who presented repeatedly were not excluded from analyses as they constitute a particularly relevant patient group that requires crisis intervention and resources. As the current paper is concerned with the sum of emergency presentations independent of whether these presentations are due to the same individuals or not, a single patient reappearing was not considered as “repeated measure.” Each presentation was considered as an independent occurance that required emergency care and resources. On average the patients received about two psychiatric diagnoses (*M* = 2.07, SD = 1.14) with the maximum number of diagnoses reaching eight in a few cases. Of the emergency presentations and including patients of the branch offices, 47.1% were admitted to the inpatient units of the hospital. In total there were 424 inpatient admissions during the observation period, with the average length of stay reaching almost 12 days (*M* = 11.62, *SD* = 28.86) as presented in Table 2.

**Table 1:** Demographic and clinical characteristics of the sample per 4th quarter over the years of observation.

	**Years of Observation**					
**Variables**	**2014**	**2015**	**2016**	**2017**	**2018**	**Total**
Number of cases	134	174	138	234	222	902
**Age (years; months)**						
M	15;5	15;7	15;6	15;2	15;5	15;4
SD	2;8	2;6	2;4	2;7	2;4	2;6
Range	1;11 – 18;4	6;5 – 19;1	8;3 – 20;5	1;10 – 19;11	4;4 – 20;4	1;10 – 20;5
Female Patients *n* (%)	78 (58.2)	87 (50.0)	73 (47.1)	132 (43.6)	151 (32.0)	521 (57.8)
**Inpatient admissions**						
Total Sample *n* (%)	58 (43.3)	89 (51.1)	70 (50.7)	111 (47.4)	96 (43.4)	424 (47.1)
Suicidal Thoughts *n* (%)	45 (53.6)	53 (58.2)	45 (60.8)	76 (59.8)	65 (57.5)	284 (58.1)
Suicide Attempts *n* (%)	8 (88.9)	10 (83.3)	5 (71.4)	3 (50.0)	6 (75.0)	32 (76.2)
NSSI *n* (%)	16 (51.6)	25 (56.8)	23 (53.5)	32 (60.4)	30 (50.0)	126 (54.5)
Compulsory Presentations (police or court) *n* (%)	10 (7.5)	29 (16.7)	21 (15.2)	23 (9.8)	39 (17.6)	122 (13.5)
**Diagnostic groups**						
F1 *n* (%)	11 (8.2)	18 (10.3)	21 (15.2)	63 (26.9)	39 (17.6)	152 (16.9)
F2 *n* (%)	3 (2.2)	1 (0.6)	7 (5.1)	2 (0.9)	1 (0.5)	14 (1.6)
F3 *n* (%)	51 (38.1)	84 (48.3)	84 (60.9)	120 (51.3)	131 (59.0)	470 (52.1)
F4 *n* (%)	65 (48.5)	85 (48.9)	57 (41.3)	82 (35.0)	100 (45.0)	389 (43.1)
F5 *n* (%)	9 (6.7)	7 (4.0)	8 (5.8)	25 (10.7)	9 (4.1)	58 (6.4)
F6 *n* (%)	8 (6.0)	22 (12.6)	8 (5.8)	12 (5.1)	14 (6.3)	64 (7.1)
F7 *n* (%)	–	–	–	–	–	–
F8 *n* (%)	2 (1.5)	7 (4.0)	4 (2.9)	2 (0.9)	2 (0.9)	17 (1.9)
F9 *n* (%)	55 (41.0)	61 (35.1)	58 (42.0)	112 (47.9)	85 (38.1)	371 (41.1)
Multiple Diagnoses *n* (%)	68 (50.7)	107 (61.5)	93 (67.4)	147 (62.8)	148 (66.7)	563 (62.4)

*NSSI, Non-suicidal Self-injury. F-diagnoses do not add up to 100 % as multiple diagnoses were possible*.

**Table 2:** Description of total sample and subsamples for duration of inpatient stay and number of psychiatric diagnoses according to ICD-10.

**Variable**	***M (SD)***	**Range**	***n***
**Duration of inpatient stay**			
Total Sample	11.62 (28.86)	0-381	423
NSSI	7.38 (12.97)	0-92	126
Suicidal Thoughts	9.36 (25.17)	0-381	284
Suicide Attempts	18.41 (24.32)	0-92	32
**Number of diagnoses**			
Total Sample	2.07 (1.14)	0-8	901
NSSI	2.06 (0.96)	1-6	231
Suicidal Thoughts	2.01 (1.14)	0−8	489
Suicide Attempts	2.02 (1.00)	0-5	42

*NSSI, Non-suicidal Self-injury*.

### Development of Numbers of Consultations Over the Years

**Figure 1:**
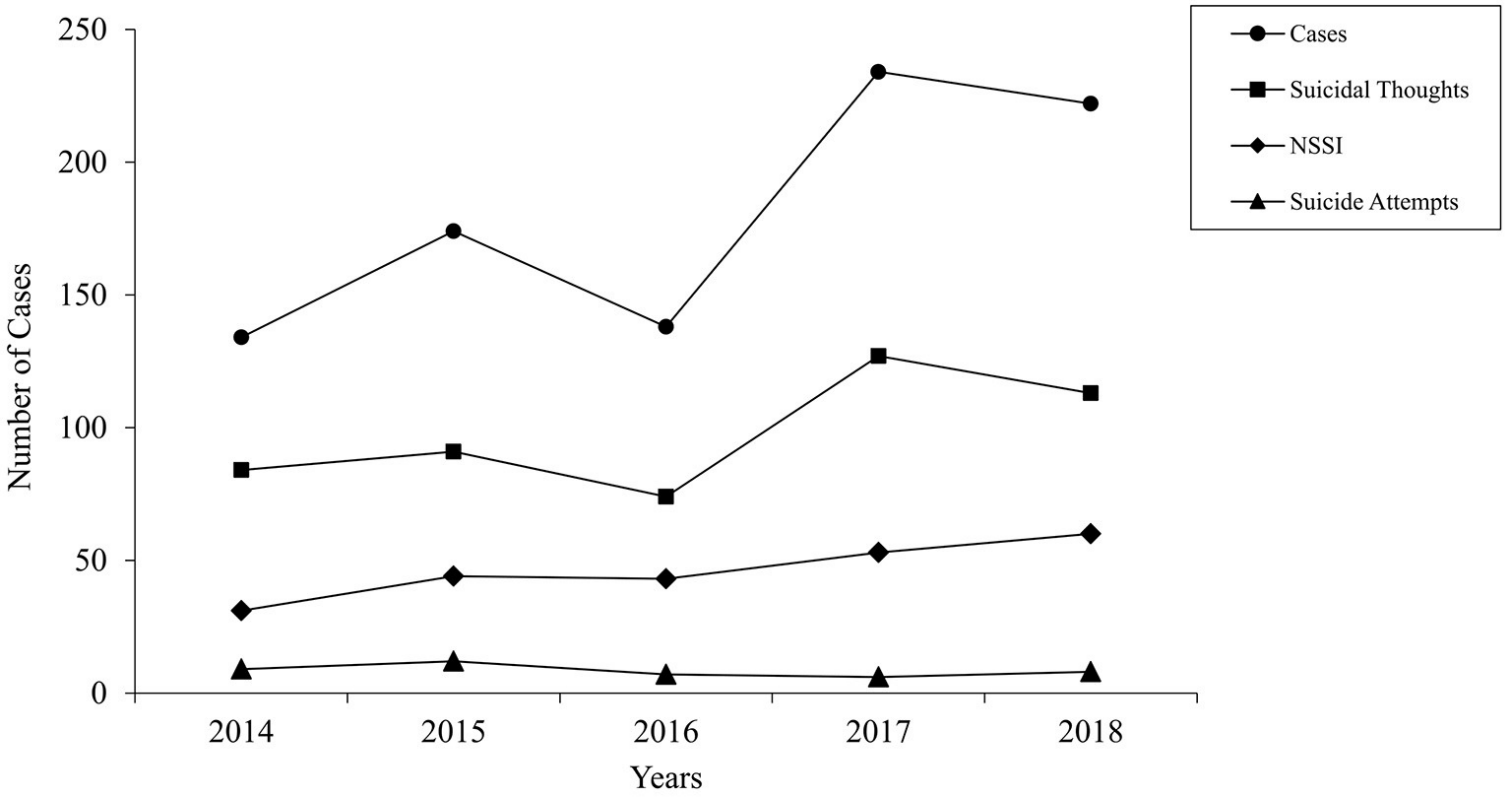
Total number of emergency presentations between 2014 and 2018, fourth quarter of each year. Emergency presentations due to suicide attempts, suicidal thoughts, and NSSI are depicted separately. NSSI, non-suicidal self-injury.

Figure 1 shows an overview of cases within each year, additionally split according to the main reasons under consideration. A significant positive correlation between time (in months) and number of emergency presentations was found [τ_(13)_ = 0.54, *p* = 0.005]. To determine the influence of time on the number of emergency presentations, a simple linear regression was computed. The model was significant [*F*_(1,13)_ = 15.70, *p* = 0.002] and explained a total of 54.7% of the variance in the dependent variable. Additionally, time was a significant predictor of the number of emergency presentations (*t* = 3.96, *p* = 0.002) (see Table 3). A closer look at suicidality and NSSI as reasons for emergency presentations revealed significant correlations between the variables time (in months) and suicidal thoughts [τ_(13)_ = 0.43, *p* = 0.028] and NSSI [τ_(13)_ = 0.48, *p* = 0.014], respectively. There was no significant relationship between suicide attempt as a reason for presentation and time [τ_(13)_ = −0.13, *p* = 0.538]. In order to analyze the influence of time on suicidal thoughts and NSSI, two simple linear regressions were performed. The regression model was significant for both, suicidal thoughts [*F*_(1,13)_ = 6.66, *p* = 0.023] and NSSI [*F*_(1,13)_ = 10.40, *p* = 0.007] and explained 33.9 and 44.5% of the variance for suicidal thoughts and NSSI, respectively. Time was a significant predictor in both cases (suicidal thoughts: *t* = 2.58, *p* = 0.023; NSSI: *t* = 3.23, *p* = 0.007). An overview of regression analyses with time as an independent variable and number of emergency presentations as well as NSSI and suicidal thoughts as reasons for emergency presentations as the dependent variables is presented in Table 3.

**Table 3:** Results of linear regression models with time predicting number of cases, NSSI, suicidal thoughts and rate of multiple diagnoses.

**Dependent variables**	**Predictor**	**B**	**SE**	**β**	**t**	**p**	**R^**2**^**
Number of cases	Time	2.54	0.64	0.74	3.96	0.002	0.55
NSSI	Time	0.74	0.23	0.67	3.23	0.007	0.45
Suicidal thoughts	Time	1.08	0.42	0.58	2.58	0.023	0.34
Rate of multiple diagnoses	Time	0.02	0.01	0.78	4.47	0.001	0.61

### Relationship of Suicidality and NSSI With Admissions and Duration of Inpatient Stay

Chi-square tests were computed to determine whether emergency presentations due to suicidal thoughts, suicide attempts or NSSI were more likely to result in inpatient admissions. All three were significant with χ^2^(1, *N* = 890) = 48.43, *p* < 0.001, χ^2^(1, *N* = 890) = 14.52, *p* < 0.001 and χ^2^(1, *N* = 890) = 6.16, *p* = 0.013, respectively. To compute the impact of reasons for emergency presentations on the inpatient stay durations, Mann-Whitney *U*-tests were computed. There was no effect of NSSI (*U* = 17,565.00, *p* = 0.315) and suicidal thoughts (*U* = 17494.00, *p* = 0.08) as a reason for the emergency presentation on inpatient stay duration, however, suicide attempts (*U* = 4539.00, *p* = 0.03) resulted in longer inpatient stays (see Table 4).

**Table 4:** Group differences in the duration of inpatient stay and number of psychiatric diagnoses for the different reasons of emergency presentations (Results of Mann-Whitney *U*-tests for dependent variables).

**Dependent variables**	**Group variable**	**Mean rank**	**U**	**p**
Duration of inpatient stay	NSSI	no = 215.86 yes = 202.91	17565.00	0.315*^a^*
	Suicidal thoughts	no = 228.14 yes = 204.10	17494.00	0.08*^a^*
	Suicide attempts	no = 207.61 yes = 265.66	4539.00	0.03*^a^*
Number of diagnoses	NSSI	no = 442.31 yes = 454.60	74013.00	0.513
	Suicidal thoughts	no = 461.69 yes = 432.22	91551.00	0.075
	Suicide attempts	no = 445.47 yes = 446.06	17784.50	0.988

*N = 890; NSSI, Non-suicidal Self-Injury*;

a *p-values have been corrected according to the False Discovery Rate (FDR)*.

### Relationship of Suicidality and NSSI With Number of Psychiatric Diagnoses or Rate of Cases With Multiple Diagnoses

There was no significant correlations between time and number of diagnoses [τ_(13)_ = −0.28, *p* = 0.151]. Mann-Whitney *U*-tests were computed to determine whether patients who gave suicidal thoughts, suicide attempts and NSSI as reasons for their emergency presentation had more diagnoses on average. None of the tests were significant, indicating no difference in the average number of diagnoses in relation to suicidal thoughts, suicide attempts and NSSI as reasons for the emergency presentation (see Table 4).

Due to the strong clinical impression that cases are becoming more complex, patients were grouped into those that had equal/more than two diagnoses (“multiple diagnoses”) and those that had <2 diagnoses (“single diagnose”). There was a significant positive correlation between time and the number of cases with multiple diagnoses [τ_(13)_ = 0.52, *p* = 0.007]. However, this relationship did not remain significant when controlling for the general number of cases in a partial correlation [*r*_(13)_ = 0.40, *p* = 0.153]. Since the strength of the relationship remained strong, a rate of cases with multiple diagnoses vs. cases with single diagnose was computed over the years, thereby determining the percentage of cases with multiple diagnoses without the bias of fluctuations in number of cases over the years. Since there was a significant correlation between time and the rate of cases with multiple diagnoses [τ_(13)_ = 0.54, *p* = 0.005], a simple lineal regression was calculated. The regression model was significant [*F*_(1,13)_ = 19.98, *p* = 0.001] and explained 61.0% of the variance in the rate of cases with multiple diagnoses. Time was a significant predictor [*t*_(13)_ = 4.47, *p* = 0.001] (see Table 3).

There was a significant correlation between cases in which suicidal thoughts and NSSI were reasons for emergency presentations and the rate of multiple diagnoses, τ_(13)_ = 0.70, *p* < 0.001 and τ_(13)_ = 0.56, *p* = 0.004, respectively. This relationship was not present for suicide attempts as a reason for the emergency presentation, τ_(13)_ = 0.00, *p* > 0.999.

## Discussion

The main purpose of this present study was to examine the frequency and possible changes in demographic and clinical characteristics of emergency outpatient presentations at our hospital over a 5-year time course. We were particularly interested in suicidality and NSSI as reasons that led to emergency presentation. In addition, we also investigated crisis-intervention inpatient admissions from emergency consultations and the number of psychiatric diagnoses among patients.

By means of a retrospective data evaluation, we analyzed the rate and changing demographic and clinical patterns of face-to-face emergency presentations at our child and adolescent psychiatric emergency service. To depict the time course over the years 2014-2018, we examined the 4th quarter of each of the determined years using the hospital's documentation system. Overall, 57.8% of the emergency presentations in our clinic were female and the average age of presentations was 15;4 years. With regard to the sex distribution and mean age during the emergency consultation a previous study in Germany (33) revealed approximately similar findings (14.5 years, 56.2% females), whereas a recent study in France (13) which captured a longer time period starting from 1981 found a younger age (13;10 years) and a slightly higher rate of male children and adolescents (aggressive behavior as a frequent reason for presentation is likely to explain the higher proportion of boys).

The increase in the development of emergency consultations is of great importance for a clinic in order to be able to estimate the demand and the challenge associated with the growing number of patients. Data from this present study showed a significant increase in the number of cases for the last years in the investigated time span (2014-2018). According to the computed regression model, each additional month resulted in an increase of 2.54 cases. A study in France has also found a constant growth in the use of emergency consultations over the study period, albeit with the greatest growth during the 1980s (13). An analysis of emergency presentations in Denmark between 2001 and 2010 demonstrated an annual average increase of 15%, from 2009 to 2010 the number of consultations had declined by 12% (14). We were able to observe this effect of slowing or reversing the trend in our survey when comparing 2017 and 2018 (see Figure 1). Here it would be informative to observe the further time trend of emergency presentations. Nevertheless, the current course shows the increasing burden to clinics for child and adolescent psychiatry. The increase in emergency presentations is challenging for clinicians, as far-reaching and difficult decisions often have to be made within a short time. In addition, it is important to be well-prepared structurally and organizationally for this clientele in order to be able to provide high quality mental health care.

We were able to confirm that for every additional month the frequency of suicidal thoughts as reason for an emergency presentation increased by 1.08 cases and the frequency of NSSI by 0.74 cases. Contrary to our expectations, suicide attempts had fortunately not increased as a reason for emergency presentation. In Denmark, a survey between 2003 and 2006 found a doubling in prevalence of suicidal ideation as the single cause of not-admitted emergency presentations (6 vs. 13.1%); suicidal ideation was present in combination with other symptoms (e.g., social problems) in about one third of emergency contacts (14). In this Danish survey, suicide attempts were also unchanged as a reason of presentation (14). A survey of pediatric psychiatric emergency department visits in 2002 found that 47% of presentations reported suicidality (alone or in combination) as a main symptom (34). Primary indication for psychiatric consultation was suicidal ideation at 39% in another survey from a pediatric emergency department in the USA (35). NSSI led to 36.6% of emergency presentations in a retrospective chart review of a clinic for child and adolescent psychiatry in Germany (33). Suicidal ideation was the most common reason for emergency presentation in our study, accounting for 54.2%. NSSI was reported as the reason for presentation in 25.6% of emergency presentations and suicide attempt was obtained as reason for presentation by 4.7%. In our study, several reasons for emergency consultations were collected and evaluated. This could partly explain deviating results. An important research topic, but one that was not included in our survey, is the issue of alexithymia and resilience. Both alexithymia and low resilience were significant predictors of increased suicidal thoughts (36). These aspects would be exciting to consider in future surveys, especially since suicidal ideation was the most common reason for emergency presentation in our study. In general, our results are in line with previous studies on this topic. Our data clearly show an increasing burden to the CAP clinics. For the treatment of suicidal thoughts and NSSI in childhood and adolescence, special interventions must be chosen that pay attention to the risk of self-endangerment of the child.

We could show that emergency presentations due to suicidal thoughts, suicide attempts or NSSI were more likely to result in an admission to the inpatient units. Furthermore, suicide attempts resulted in longer inpatient stays. Former findings from another clinic for child and adolescent psychiatry in Germany showed that half of the crisis admissions were at risk of suicide at the time of admission, while this was the case in only 5.1% of the planned admissions (15). The results for self-injurious behavior were not quite as clear, but similar; self-injury was present in 56.1% of crisis admissions, in planned admissions, this was the fact in 30.9% (15). The previous study showed a decrease in inpatient stay duration for crisis admissions from 40.4 days (2005) to 19.2 days (2015) (15). However, between 2005 and 2015, there was an increase of 571% in treatment episodes lasting <10 days (15). Thus, before 2009, most patients admitted in crisis almost automatically received regular treatment on the occasion of their crisis, whereas in 2015 the majority of patients received only crisis-focused treatment (15). We explain our finding of longer inpatient stays by the fact that inpatient admissions after psychiatric emergency consultations due to suicide attempts require a respective duration of inpatient treatment, which can hardly be limited further. Most patients admitted to our clinic for crisis intervention are also treated with a crisis focus. However, care must also be taken to ensure that patients are not discharged too quickly and that sufficient intervention and improvement has occurred that readmissions are not required.

The hypothesis that the presence of a greater amount of number of psychiatric diagnoses increases over time could not be confirmed. In further exploratory analyses the data was split according to cases with two or more diagnoses (“multiple diagnoses”) and cases with single diagnosis. A rate of multiple diagnoses was computed in order to avoid the bias of generally increasing number of cases and observe multiple diagnoses in relation to single diagnoses. In this reduced data, a positive association between time and the rate of multiple diagnoses was found, indicating that cases with multiple diagnoses increased independent of the general increase in emergency presentations. This increasing number of emerging cases constitutes a constantly growing challenge for the clinic, in terms of personnel, structure and organization. Either, it suggests that additional psychiatric disorders will have to be examined diagnostically in the future, especially since the emergency presentation is partly the gateway to child and adolescent psychiatric treatment (8, 9). Or it suggests that the burden of multiple diagnoses on patients is likely to be increased and, accordingly, multiple aspects need to be considered in the emergency consultation. This in turn can make the evaluation of the crisis situation more difficult. Multiple psychiatric diagnoses often manifest themselves in higher symptom severity and also have to be taken into account in following treatment (29, 30). In the overall spectrum of self-harming behaviors, the context of substance use should not be neglected. Past research has shown a strong relationship between suicidality and substance use, especially among adolescents (25–27). In a study in France, substance use disorder as a main psychiatric diagnosis was four times more common among youth admitted to the emergency department in 2017 compared to 1992 (13). In our sample, the overall proportion of patients with an F1 diagnosis was 16.9%, whereby we surveyed all available diagnoses and not only the main diagnosis. The proportion of F1 diagnoses among emergency presentations with suicidal thoughts were 11.5% whereas a total of 37.6% of individuals with F1 diagnoses presented with suicidal thoughts. This was not examined in detail as the main question was concerned with suicidality independent of specific diagnoses. However, from our point of view, accompanying substance disorders should be taken into account in the crisis intervention of adolescents. Despite providing an extensive overview of clinic emergency presentations and detailed numbers specifically relating to suicidality and NSSI as reasons for presentations, this study has several limitations. Because this is a retrospective analysis of medical records, the data were not originally collected for research purposes, so we may have underestimated or overestimated certain patient characteristics because of differences in data availability (such as differences in documentation by treating physicians). Our study reports emergency presentations from a maximum-care child and adolescent psychiatric hospital in Germany. It may be that our data cannot be generalized to other pediatric or CAP-settings or hospitals.

Nevertheless, especially with the analysis of over 900 emergency presentations, we have achieved a remarkable sample size and results indicate specific profiles in children and adolescent with suicidality. The daily care of patients presenting to a psychiatric emergency service who require outpatient care or, if necessary, crisis intervention inpatient care, has a high priority in child and adolescent psychiatric clinics. In this context, the risk assessment and management of suicidal crises remain an important and urgent task. All centers providing care should adapt to this topic in terms of content and organization and establish special intervention and treatment programs. In addition, future research should also examine the effectiveness of crisis intervention as it is currently offered in child and adolescent psychiatric clinics.

## Data Availability Statement

The raw data supporting the conclusions of this article will be made available by the authors, without undue reservation.

## Ethics Statement

The studies involving human participants were reviewed and approved by institutional examination board for the Medical Faculty of the University of Regensburg (Ethics Commission No.: 19-1428-104). Written informed consent from the participants' legal guardian/next of kin was not required to participate in this study in accordance with the national legislation and the institutional requirements.

## Author Contributions

SK and RB had the idea for the study and developed the study design. MW conducted the retrospective data collection. The first draft was written by SK with support from AE, DS, IJ, and MW. All authors read and approved the final manuscript.

## Conflict of Interest

The authors declare that the research was conducted in the absence of any commercial or financial relationships that could be construed as a potential conflict of interest.
